# Risk factors for carbapenem-resistant *Acinetobacter baumannii* (CRAB) infections in critically ill patients with previous CRAB colonization: a multicentre cohort study

**DOI:** 10.1093/jacamr/dlaf262

**Published:** 2026-01-23

**Authors:** Francesco Cogliati Dezza, Belén Gutiérrez-Gutiérrez, Giusy Tiseo, Sara Covino, Flavia Petrucci, Jose Bravo-Ferrer, Valentina Galfo, Aurelio Lepore, Federica Sacco, Agnese Viscido, Giancarlo Ceccarelli, Francesco Alessandri, Claudio Maria Mastroianni, Mario Venditti, Marco Falcone, Jesús Rodríguez-Baño, Alessandra Oliva

**Affiliations:** Department of Public Health and Infectious Diseases, Sapienza University of Rome, Rome, Italy; Unidad Clínica de Enfermedades Infecciosas y Microbiología, Hospital Universitario Virgen Macarena and Departamento de Medicina, Universidad de Sevilla, Instituto de Biomedicina de Sevilla/CSIC, Seville, Spain; Centro de Investigación Biomédica en Red en Enfermedades Infecciosas (CIBERINFEC), Instituto de Salud Carlos III, Madrid, Spain; Unidad Clínica de Enfermedades Infecciosas y Microbiología, Hospital Universitario Virgen Macarena and Departamento de Medicina, Universidad de Sevilla, Instituto de Biomedicina de Sevilla/CSIC, Seville, Spain; Centro de Investigación Biomédica en Red en Enfermedades Infecciosas (CIBERINFEC), Instituto de Salud Carlos III, Madrid, Spain; Infectious Diseases Unit, Department of Clinical and Experimental Medicine, Azienda Ospedaliera Universitaria Pisana, University of Pisa, Pisa, Italy; Department of Public Health and Infectious Diseases, Sapienza University of Rome, Rome, Italy; Department of Public Health and Infectious Diseases, Sapienza University of Rome, Rome, Italy; Unidad Clínica de Enfermedades Infecciosas y Microbiología, Hospital Universitario Virgen Macarena and Departamento de Medicina, Universidad de Sevilla, Instituto de Biomedicina de Sevilla/CSIC, Seville, Spain; Centro de Investigación Biomédica en Red en Enfermedades Infecciosas (CIBERINFEC), Instituto de Salud Carlos III, Madrid, Spain; Infectious Diseases Unit, Department of Clinical and Experimental Medicine, Azienda Ospedaliera Universitaria Pisana, University of Pisa, Pisa, Italy; Infectious Diseases Unit, Department of Clinical and Experimental Medicine, Azienda Ospedaliera Universitaria Pisana, University of Pisa, Pisa, Italy; Microbiology and Virology Laboratory, Department of Molecular Medicine, Sapienza University of Rome, Rome, Italy; Microbiology and Virology Laboratory, Department of Molecular Medicine, Sapienza University of Rome, Rome, Italy; Department of Public Health and Infectious Diseases, Sapienza University of Rome, Rome, Italy; Department of General and Specialistic Surgery, Sapienza University of Rome, Rome, Italy; Department of Public Health and Infectious Diseases, Sapienza University of Rome, Rome, Italy; Department of Public Health and Infectious Diseases, Sapienza University of Rome, Rome, Italy; Infectious Diseases Unit, Department of Clinical and Experimental Medicine, Azienda Ospedaliera Universitaria Pisana, University of Pisa, Pisa, Italy; Unidad Clínica de Enfermedades Infecciosas y Microbiología, Hospital Universitario Virgen Macarena and Departamento de Medicina, Universidad de Sevilla, Instituto de Biomedicina de Sevilla/CSIC, Seville, Spain; Centro de Investigación Biomédica en Red en Enfermedades Infecciosas (CIBERINFEC), Instituto de Salud Carlos III, Madrid, Spain; Department of Public Health and Infectious Diseases, Sapienza University of Rome, Rome, Italy

## Abstract

**Background:**

Among MDR bacteria, carbapenem-resistant *Acinetobacter baumannii* (CRAB) is a major concern due to the limited therapeutic options.

**Objectives:**

To identify predictors to aid in the clinical management of critically ill patients.

**Methods:**

We conducted a multicentre prospective study in Italy, enrolling patients with CRAB colonization who were admitted to ICUs between 2020 and 2023. Multivariable logistic regression analysis was performed to identify potential risk factors for CRAB infection. To account for competing risks, we used the cumulative incidence function (CIF) and Fine–Gray regression analysis, providing an accurate assessment of the risk of CRAB infection. Additionally, a logistic regression model was performed to estimate the impact of different types of critically ill patients on the risk of infection.

**Results:**

We included 564 colonized patients, and 381 (67.5%) developed a CRAB infection in the ICU. In the logistic regression model, multisite colonization (OR 2.78; 95% CI: 1.90–4.08; *P* < 0.001), Charlson comorbidity index (CCI) ≥3 (OR 1.59; 95% CI: 1.00–2.50; *P* = 0.047), mechanical ventilation (OR 1.48; 95% CI: 1.00–2.18; *P* = 0.048), male gender (OR 2.06; 95% CI: 1.38–3.10; *P* < 0.001), and time from ICU admission to colonization ≤12 days (OR 2.00; 95% CI: 1.36–2.94; *P* < 0.001) were independent predictors of CRAB infection. Findings were confirmed in the Fine–Gray model. In a secondary model, COVID-19 (OR 2.31; 95% CI: 1.30–4.10; *P* = 0.004) and burn patients (OR 4.84; 95% CI: 1.65–14.17; *P* = 0.004) were risk factors for CRAB infection.

**Conclusions:**

Early colonization from ICU admission, multisite colonization, CCI, mechanical ventilation and male gender are key risk factors for CRAB infection. These factors support clinicians in the management of critically ill patients with prior CRAB colonization.

## Introduction

Antimicrobial resistance is a ‘silent’ pandemic threatening public health worldwide.^1^ Patients admitted to ICUs are at high risk of infection caused by MDR microorganisms because of exposure to several predisposing factors, such as underlying clinical severity, prolonged hospitalization, high antibiotic pressure and invasive procedures.

Carbapenem-resistant *Acinetobacter baumannii* (CRAB) is one of the microorganisms included in the WHO’s critical priority list.^2^ Despite the recent approval of new antibiotics active *in vitro* against MDR Gram-negative bacteria, most are not active against CRAB, and therefore CRAB infections are still associated with up to 60% mortality.^3–5^ Moreover, the COVID-19 pandemic has led to a rise in CRAB infections in some hospitals, with an important impact on in-hospital mortality.^6,7^

Appropriate antibiotic therapy should be started promptly in critically ill patients to improve their outcomes. But, at the same time, overuse of antibiotics must be avoided to reduce the spread of antimicrobial resistance. To address these needs, clinical scores have been proposed to guide empirical antibiotic therapy in these difficult-to-treat infections. Previous colonization is frequent in patients developing CRAB infection. Differently from carbapenem-resistant Enterobacterales, for which several well-established clinical risk scores exist, predictive factors for CRAB infections in previously colonized patients have been investigated in only a few studies.^8–11^ Knowing the specific risk factors for infection development in critically ill patients colonized by CRAB might be crucial in prompting early appropriate therapy aimed at reducing associated mortality, as reported in other severe infections.^12–14^

The primary objective of this study was to investigate the risk factors for developing a CRAB infection in ICU patients with prior CRAB colonization. The secondary objective was to assess the impact of different types of critically ill patients in the development of CRAB infection.

## Materials and methods

### Study design and data

This study was a prospective, observational multicentre study in adult patients admitted to ICUs in two tertiary academic hospitals in Italy, located in Rome and Pisa. This cohort included all consecutive adult patients admitted to the participating ICUs and colonized by CRAB at any anatomical site during 2020–2023. Exclusion criteria were: an expected survival <48 h from colonization, CRAB infection pre-ICU admission, and colonization detected >30 days previous to ICU admission. Patients who developed CRAB infections without previous colonization were not included. STROBE recommendations were followed for study reporting (Table S1, available as Supplementary data at *JAC-AMR* Online).

### Setting and study variables

At the time of the study, the hospital in Rome had one general ICU, one dedicated to the emergency room (ER-ICU), one dedicated to neurosurgery (NS-ICU), one dedicated to cardiothoracic surgery (CTS-ICU), one devoted to organ transplant and one devoted to COVID-19, as previously reported.^11^ The hospital in Pisa included similar units, with an additional one dedicated to burn patients. According to local infection control guidelines, during the study period, a systemic surveillance protocol was implemented in the participating ICU: rectal/stool swabs, respiratory and urine cultures were performed at ICU admission and routinely re-evaluated once weekly (in Rome) or twice weekly (in Pisa) for detection of MDR organism (MDRO) strains. In the burn unit also skin swabs were performed routinely. In cases of CRAB colonization outside the ICU before admission, systematic MDRO screening was not performed unless indicated for clinical or epidemiological reasons.

Trained infectious disease specialists revised medical records, and the following information was anonymously recorded in an electronic database: demographics; comorbidities; hospitalization/antibiotic therapy/immunosuppression/surgery in the previous 90 days; cause of hospitalization and ICU admission; type of ICU; laboratory and clinical data on the day of colonization; source of infection; invasive procedures; site of colonization; antibiotic therapy; timing of colonization and infection onset; length of stay in ICU and in hospital; in-hospital mortality; and 7 day, 14 day and 28 day mortality from colonization and infection onset. Patients were followed until hospital discharge or in-hospital mortality. The burden of comorbidities was estimated by means of the Charlson comorbidity index (CCI), whereas patients’ severity at ICU admission was defined by the Simplified Acute Physiology Score II (SAPS II).^15,16^ Immunosuppression was defined as steroid therapy with prednisone (or its equivalent) at a dose of >0.5 mg/kg/day for at least 1 month or the receipt of chemotherapy, TNF-α inhibitors, cyclophosphamide, azathioprine, methotrexate or mycophenolate mofetil in the previous 90 days.

### Endpoint and definitions

The primary endpoint was any type of CRAB infection during the hospitalization after CRAB colonization. Furthermore, we assessed the impact of the different types of ICU patients in the development of CRAB infection.

Colonized-only patients were patients with CRAB colonization who did not develop CRAB infection during the entire hospitalization. Patients with infection were those who developed a CRAB infection during hospitalization. CRAB infections were identified when CRAB was isolated from a clinical specimen in the presence of clinical signs and symptoms of infection, following the standard definitions provided by the ECDC for healthcare-related infections.^17^ The date of infection onset was defined as the date of the index specimen collection.

Colonization was defined as the isolation of CRAB from screening samples or from any clinical sample without fulfilling the criteria for infection. All patients underwent CRAB screening at ICU admission (within 48 h) according to local infection-control protocols, and baseline colonization status was defined using this admission screen. Patients with a positive CRAB screening result within the first 48 h of ICU stay were considered colonized at ICU admission. For all other patients, time to colonization was calculated as the number of days from ICU admission to the first positive CRAB screening sample during the ICU stay. The differences between CRAB colonization and infection, in sites other than rectal swab isolation, were evaluated case by case; in case of doubt, a decision was taken after discussion among investigators. Multisite colonization was defined as the isolation of CRAB from more than one specimen from different anatomical sites in the same patient during ICU hospitalization; repeat isolates from the same site were counted only once.

### Microbiological studies

The identification of CRAB strains was based on local laboratory techniques. Blood cultures were incubated in the automatic BacT/ALERT Virtuo system (bioMérieux, Inc., Marcy l’Étoile, France). Positive blood cultures of Gram-negative bacteria and other surveillance samples (respiratory secretions, rectal swab) were cultured on agar media (blood agar, MacConkey agar, bioMérieux, Marcy l’Étoile, France; Brilliance CRE agar, Thermo Fisher Scientific) and incubated for 24 h at 37°C. The bacterial colonies were identified by MALDI-TOF MS system (Bruker Daltonik GmbH, Bremen, Germany), with a discriminatory score >2300. Antimicrobial susceptibility profiles were tested using the MicroScan WalkAway system (Beckman Coulter, Inc., Brea, CA, USA). Antimicrobial susceptibility testing was interpreted according to EUCAST clinical breakpoints.^18^

### Statistical analysis

Categorical variables were presented as frequency counts with percentages. Continuous variables were presented as median with IQRs. Continuous variables were compared using Student’s *t*-test or Mann–Whitney *U*-test, as appropriate. Categorical variables were compared using chi-squared or Fisher’s exact test, as appropriate.

Continuous and polychotomous variables were dichotomized using classification and regression trees (CART) analysis according to their association with CRAB infection onset (Figure S1). The interactions between variables and the main outcome were also explored through CART analysis and Youden’s index. Missing data are reported; variables with >20% missing data were excluded from multivariate analysis (as shown in Table 1, variables with >20% missing data reported were SAPS II, last negative screening, previous antibiotic exposure with new beta lactam-beta lactamase inhibitor).

**Table 1.** General population and colonized-only patients versus patients with infection^a^

Characteristics	General population *N* = 564	Colonized-only patients *N* = 183	Patients with infection *N* = 381^e^	OR (95% CI)	*P* value
**General, *n* (%)**					
Centre, Rome/Pisa	292 (50.8)/272 (49.2)	106 (57.9)/77 (42.1)	186 (48.8)/165 (51.2)	1.44 (1.01–2.06)	**0**.**043**
Age, median (IQR), y	64 (51–74)	65 (49.5–74)	64 (53–74)		0.550
Gender male	406 (72)	116 (63.4)	290 (76.1)	0.54 (0.37–0.80)	**0**.**002**
Type of ICU patient					**<0.001** ^f^
General unspecified	99 (17.6)	40 (21.9)	59 (15.5)	0.65 (0.42–1.02)	
COVID-19	167 (29.6)	41 (22.4)	126 (33.1)	1.71 (1.14–2.57)	
Neurosurgery	82 (14.5)	44 (24)	38 (10)	0.35 (0.22–0.57)	
Transplant	9 (1.6)	3 (1.6)	6 (1.6)	0.96 (0.29–3.9)	
Cardiac surgery	21 (3.7)	7 (3.8)	14 (3.7)	0.96 (0.38–2.42)	
Emergency room patients	136 (24.1)	43 (23.5)	93 (24.4)	1.05 (0.69–1.59)	
Burns	50 (8.9)	5 (2.7)	45 (11.8)	4.77 (1.88–12.34)	
**Comorbidities, *n* (%)**					
Myocardial infarction	113 (20.1)	19 (10.4)	94 (24.7)	2.81 (1.65–4.77)	**<0**.**001**
Congestive heart failure	109 (19.4)	22 (12.1)	87 (22.8)	2.15 (1.3–3.57)	**0**.**003**
Diabetes mellitus	96 (17.1)	29 (15.9)	67 (17.6)	1.16 (0.70–1.81)	0.626
Chronic kidney disease^c^	32 (5.7)	5 (2.7)	27 (7.1)	2.84 (1.08–7.13)	**0**.**038**
Solid tumour	72 (12.8)	24 (12.4)	48 (13)	0.95 (0.6–1.70)	0.845
Haematological malignancies	22 (3.9)	7 (3.8)	15 (3.9)	1.02 (0.40–2.29)	0.936
Solid organ transplant	5 (0.9)	3 (1.6)	2 (0.5)	0.32 (0.05–1.92)	0.336^d^
Charlson comorbidity index, median (IQR)	1 (0–2)	1 (0–2)	1 (0–3)	—	0.196
Cut-off value ≥3, *n* (%)	135 (24)	35 (19.2)	100 (26.2)	1.49 (0.97–2.31)	0.068
SAPS II score, median (IQR)^b^	40 (31–52)	37.5 (29–53)	41 (33–51)	—	0.214
**CRAB colonization, *n* (%)**					
First detection of colonization in ICU	553 (98)	180 (98.4)	373 (97.9)	0.78 (0.20–2.96)	0.711
Timing of colonization, median (IQR), d					**0.023**
From ER admission	13 (7–21)	15 (7–25)	6 (12–20)	**—**	**0.019**
From ICU admission	10 (5–16)	11 (5–22)	9 (5–15)	**—**	**0.002**
Cut-off value ≤12 days, *n* (%)	351 (62.2)	97 (53)	254 (66.7)	1.77 (1.24–2.54)	0.093
From last negative screening^b^	4 (3–5)	4 (3–6)	4 (3–4)	**—**	
Site of colonization					
Respiratory tract	351 (62.2)	98 (53.6)	253 (66.4)	1.72 (1.2–2.45)	**0.003**
Rectal swab	458 (81.2)	142 (77.6)	316 (81.2)	1.44 (0.91–2.22)	0.128
Urine	55 (9.8)	14 (7.7)	41 (10.8)	1.51 (0.8–2.85)	0.244
Skin and soft tissue	50 (8.9)	6 (3.3)	44 (11.5)	3.89 (1.63–9.3)	**0.001**
Drainage	2 (0.3)	0 (0)	2 (0.5)	—	1.0^d^
CVC	21 (3.7)	5 (2.7)	16 (4.1)	1.58 (0.57–4.38)	0.376
Multisite >1	310 (55)	72 (39.3)	238 (62.5)	2.66 (1.79–3.68)	**<0**.**001**
Number of sites, median (IQR)	2 (1–2)	1 (1–2)	2 (1–2)		**<0.001**
2	247 (44.2)	62 (34.3)	185 (49)	1.84 (1.33–2.76)	**<0.001**
3	56 (10)	6 (3.3)	50 (13.2)	4.49 (1.89–10.68)	**<0.001**
Colonization by other MDRO	239 (42.4)	82 (44.8)	157 (41)	0.82 (0.58–1.17)	0.287
Type of ventilation					**0.006**
No oxygen	83 (14.8)	39 (21.2)	44 (11.7)		
VMK	190 (34)	63 (34.2)	127 (33.9)		
NIV	31 (5.5)	13 (7.1)	18 (4.8)		
Mechanical ventilation	244 (43.3)	64 (35)	180 (47.2)	1.64 (1.16–2.40)	**0.006**
ECMO	34 (6.6)	9 (5.2)	25 (7.3)	1.42 (0.65–3.12)	0.375
CRRT	38 (7.4)	8 (4.7)	30 (8.7)	1.96 (0.88–4.38)	0.094
Antibiotic therapy before colonization	467 (82.8)	155 (84.7)	312 (81.9)	0.82 (0.51–1.32)	0.408
BL-BLI use	341 (73.2)	116 (76.3)	223 (71.7)	0.29 (0.79–1.23)	0.226
New BL-BLI use^b^	34 (8.3)	22 (16.9)	12 (4.3)	0.22 (0.10–0.46)	**<0.001**
Days of antibiotic therapy					
<7	203 (44.8)	56 (37.3)	147 (48.2)	1.53 (1.02–2.28)	**0.038**
>7 and <14	124 (27.4)	38 (26.7)	86 (28.2)	1.14 (0.70–1.77)	0.572
>14	126 (27.5)	54 (36)	72 (23.4)	0.54 (0.35–0.82)	**0.004**
Steroids before colonization	244 (42.7)	81 (43.1)	161 (42.5)	0.98 (0.69–1.39)	0.892
Days of steroids, median (IQR)	9 (4–15)	10 (3–15)	9 (5–15)	—	0.957
Other immunosuppressive drugs	32 (5.7)	11 (6)	21 (5.5)	0.93 (0.45–1.9)	0.81
Central venous catheter	534 (94.7)	170 (92.8)	364 (95.5)	1.62 (0.77–3.40)	0.191
Vesical catheter	559 (99.1)	182 (99.5)	377 (99)	0.51 (0.06–4.61)	1.0
Drainage	101 (21)	37 (24.5)	64 (19.4)	0.74 (0.47–1.17)	0.202
Total parenteral nutrition	347 (61.5)	111 (60.6)	236 (61.9)	1.1 (0.77–1.58)	0.597
**Outcomes, median (IQR)**					
Hospitalization duration, d	41 (25–67)	36 (23–59)	44 (27–69)		**0**.**011**
ICU hospitalization duration, d	30 (18–49)	23 (14–40)	33 (21–52)		**<0**.**001**
In-hospital all-cause mortality, *n* (%)	274 (48.6)	60 (32.8)	274 (56.2)	2.63 (1.82–3.8)	**<0**.**001**
Mortality from colonization, *n* (%)					0.867
7 day mortality	69 (12.2)	23 (12.6)	46 (12.6)	0.95 (0.56–1.63)	0.122
14 day mortality	117 (20.7)	31 (16.9)	86 (22.6)	1.43 (0.91–2.25)	**0.003**
28 day mortality	183 (32.4)	44 (24)	139 (36.5)	1.81 (1.22–2.70)	
Time to death from colonization, d	19 (7–36)	14 (4–30)	19 (9–36)	—	**0**.**046**

Bold values indicate statistical significance.

BL-BLI, beta lactam-beta lactamase inhibitor as ampicillin/sulbactam, amoxicillin/clavulanic acid, and piperacillin/tazobactam; CRAB, carbapenem-resistant *Acinetobacter baumannii*; CRRT, continuous renal replacement therapy; CVC, central venous catheter; ECMO, extra-corporeal membrane oxygenation; ER, emergency room; new BL-BLI, meropenem/vaborbactam, ceftolozane/tazobactam, ceftazidime/avibactam; NIV, non-invasive ventilation; SAPS II score, Simplified Acute Physiology Score II; VMK, venturi mask.

^a^Data are number of patients (percentage) except where specified.

^b^Variables with more than 20% missing data were: SAPS II (*n* = 292), last negative screening (*n* = 357), and previous exposure to new BL-BLI (*n* = 412).

^c^From moderate chronic kidney disease (creatinine >3 mg/dL) to dialysis or status post kidney transplant.

^d^Fisher’s test.

^e^Among the 381 patients with CRAB infection, the distribution of infection types was as follows: bloodstream infection *n* = 166 (43.5%), ventilator-associated pneumonia *n* = 136 (35.7%), hospital-acquired pneumonia *n* = 40 (10.5%), urinary tract infection *n* = 13 (3.4%), complicated intra-abdominal infection *n* = 10 (2.6%), and other sites (CNS or skin and soft-tissue infections) *n* = 16 (4.3%).

^f^Global *P* value for type of ICU patients (χ² test).

To explore the independent predictors of CRAB infection, logistic regression was used; variables with a univariable *P* value <0.1 were introduced in the model and selected using a backward stepwise procedure. Continuous variables were dichotomized. Interaction effects and multicollinearity between variables were considered.

CRAB infection-free survival curves were plotted using the Kaplan–Meier method and compared using the log-rank test. The variable time-to-event in this curve was considered from the date of the first colonization to CRAB infection comparing early (≤12 days) and late (>12 days) colonization. Censoring was considered both for death or ICU discharge in patients without infection.

We built a multivariable logistic regression model for CRAB infection. Model discrimination was assessed using the area under the receiver operating characteristic curve (AUROC) with 95% CIs. The goodness of fit for observed data of the logistic regression multivariable model was assessed using the Hosmer–Lemeshow test. Calibration of the multivariable logistic regression model was assessed graphically by plotting observed versus predicted risk of CRAB infection across deciles of predicted probability (calibration plot). Observed risk in each decile was calculated as the proportion of patients who developed CRAB infection.

To account for the competing risk of death or ICU discharge, we also fitted a Fine–Gray subdistribution hazards model for time from colonization to CRAB infection; results from this model are reported as subdistribution hazard ratios (sHRs) with 95% CIs. The Fine–Gray model was assessed for suitability using Harrell's concordance index (C-index), which evaluates the model’s discriminative ability in the presence of competing risks. *P* value analyses were two-sided, and a *P* value of <0.05 was considered statistically significant.

All statistical analyses were performed with Statistical Program for the Social Science software (SPSS, version 29; SPSS Inc., Chicago, IL, USA), R software version 4.4.1 (R Foundation for Statistical Computing) and CART (Salford Predictive Modeler, version 8.0, Minitab).

### Ethics

The study was approved by the local Ethical Committees (no. 0341/2023 in Rome, no. 24111 in Pisa), and informed consent was waived due to the observational nature of the research.

## Results

### Study population

**Figure 1.**
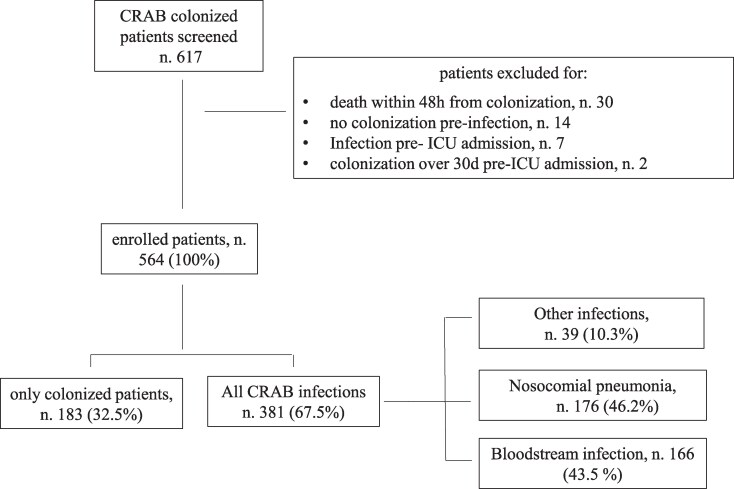
Flow chart of study population. CRAB, carbapenem-resistant *Acinetobacter baumannii*.

Overall, 617 patients were detected as colonized by CRAB; 53 had exclusion criteria, and therefore 564 were included (Figure 1). Of them, 381 (67.5%) developed CRAB infection. The median age was 64 years (IQR 51–74) and 406 (72%) were men. General characteristics are described in Table 1 and Table S2. Among the causes of ICU admission, the most common was respiratory failure (202 patients, 35.8%), followed by trauma (121, 21%) and post-surgery (105, 18.6%). The median time from ICU admission to CRAB colonization was 10 days (IQR 5–16). Almost all patients developed colonization in ICU (98%); 310 (55%) showed a multisite colonization, and the most common site of colonization was rectal followed by respiratory tract.

The overall 28 day mortality from colonization date was 32.4% [24% and 36.5% in the colonized-only and patients with infection, respectively; OR 1.81 (95% CI: 1.22–2.70), *P* = 0.03].

### Patients with CRAB infection

**Figure 2.**
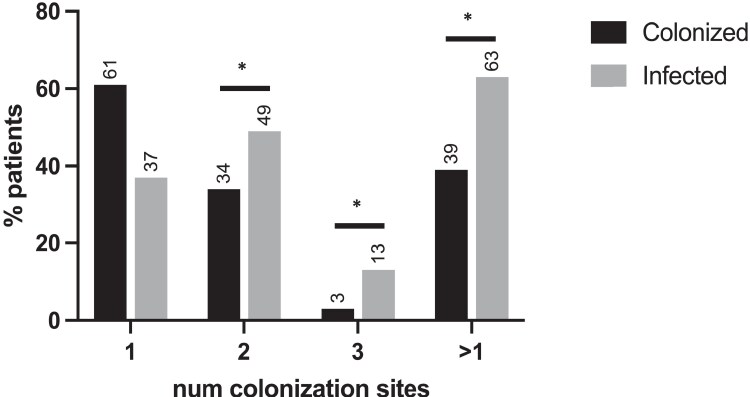
Colonization characteristics in the study population. Proportions of colonized or infected patients with one or more different anatomical colonization sites. Numbers refer to the total number of colonized or infected patients. >1:multisite colonization; *P < 0.05.

The general characteristics of patients with infection are described in Table 1. Nosocomial pneumonia (hospital-acquired or ventilator-associated) was the most frequent type of infection (176, 46.2%), followed by bloodstream infection (BSI) (166, 43.5%). Figure 2 shows the differences between colonized-only patients and those with infection in terms of colonization in different anatomical sites; multisite was present in 72 (39.3%) and 238 (62.5%) in colonized-only patients and patients with infection, respectively (OR 2.66; 95% CI: 1.79–4.38; *P* < 0.001), whereas two sites were reported in 62 (34.3%) and 185 (49%) patients (OR 1.84; 95% CI: 1.33–2.76; *P* < 0.001), and three sites in 6 (3.3%) and 50 (13.2%) patients (OR 4.49; 95% CI: 1.89–10.68; *P* < 0.001). In patients with infection, the median time from colonization to infection was 3 days (IQR 0–7).

### CRAB infection risk factors

Univariable analysis between the two groups (Table 1) showed that patients with infection more frequently had myocardial infarction (OR 2.81; 95% CI: 1.65–4.77; *P* < 001), congestive heart failure (OR 2.15; 95% CI: 1.3–3.57; *P* = 0.003), chronic kidney disease (OR 7.37; 95% CI: 0.95–57.27; *P* = 0.039), COVID-19 (OR 2.01; 95% CI: 1.35–2.98; *P* < 001), and shorter time from hospital and ICU admission to colonization (*P* = 0.023 and *P* = 0.019, respectively). On the colonization day, patients with infection were more likely to be on mechanical ventilation (OR 1.64; 95% CI: 1.16–2.4; *P* = 0.006) and have multisite colonization, as reported above.

**Figure 3.**
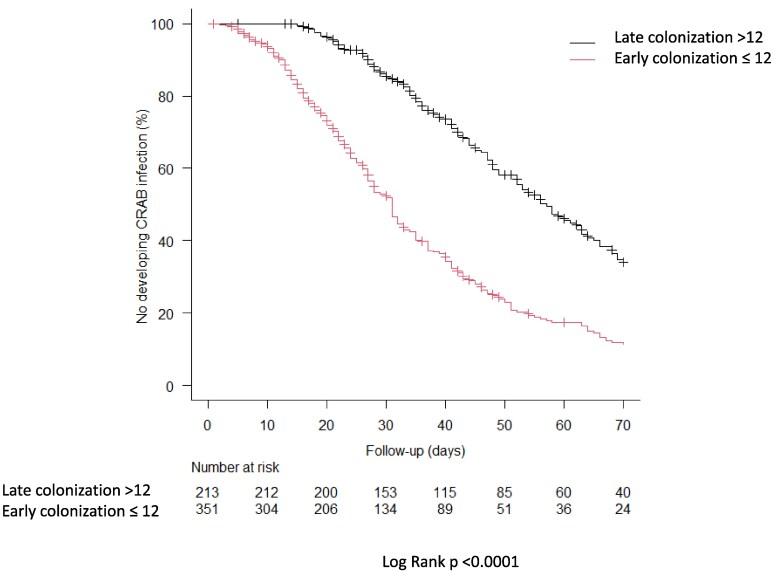
CRAB infection-free survival curve. Kaplan–Meier curves of CRAB infection-free survival between patients colonized within 12 days (early colonization patients, red line) and after 12 days (late colonization patients, black line). Patients were censored both for death or ICU discharge if no infection occurred. CRAB, carbapenem-resistant *Acinetobacter baumannii*.

The logistic regression multivariable analysis showed that multisite colonization (OR 2.78; 95% CI: 1.90–4.08; *P* < 0.001), CCI ≥3 (OR 1.59; 95% CI: 1.00–2.50; *P* = 0.047), mechanical ventilation (OR 1.48; 95% CI: 1.00–2.18; *P* = 0.048), male gender (OR 2.06; 95% CI: 1.38–3.10;, *P* < 0.001), and time from ICU admission to colonization ≤12 days (OR 2.00; 95% CI: 1.36–2.94; *P* < 0.001) were independently associated with CRAB infection (Table 2). No clinically relevant interactions were detected. Patients with an early colonization (≤12 days) had a higher cumulative proportion of infection (log-rank test *P* < 0.001), as shown in the Kaplan–Meier curve (Figure 3). Table S3 shows the impact of centre-specific screening strategy on the development of CRAB infection.

**Table 2.** Multivariable analysis for CRAB infection by logistic regression

	OR (95% CI)	*P* value^a^
Multisite colonization^b^	2.78 (1.90–4.08)	**<0.001**
Charlson comorbidity index ≥3	1.59 (1.00–2.50)	**0.047**
Mechanical ventilation^c^	1.48 (1.00–2.18)	**0.048**
Male gender	2.06 (1.38–3.10)	**<0.001**
Timing to colonization ≤12 d^d^	2.00 (1.36–2.94)	**<0.001**

AUROC 0.70 (95% CI: 0.65–0.75); Hosmer–Lemeshow test: 0.359.

^a^Bold values indicate statistical significance.

^b^Patients with CRAB colonization from more than one specimen from different anatomical sites.

^c^At the time of colonization.

^d^Time from ICU admission to CRAB colonization.

### Competing risk assessment

**Figure 4.**
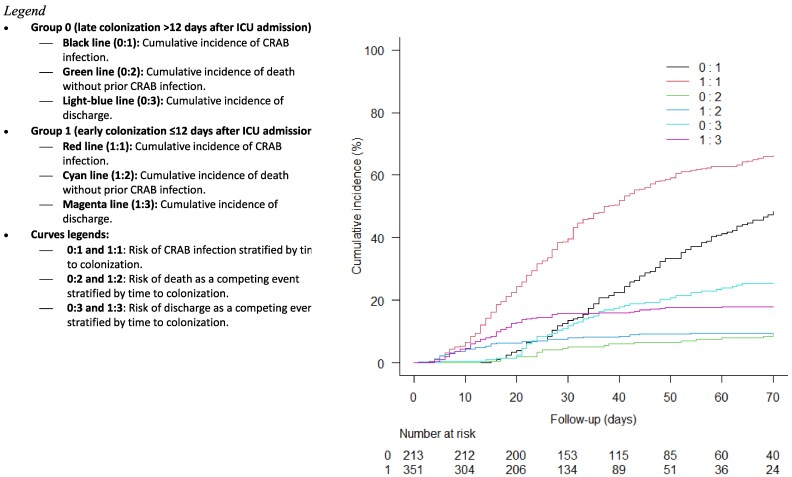
Cumulative incidence of CRAB infection and competing events based on time to colonization (early colonization ≤12 days versus late colonization >12 days). CRAB, carbapenem-resistant *Acinetobacter baumannii*.

The cumulative incidence analysis, shown in Figure 4, revealed that patients colonized within 12 days of ICU admission had a significantly higher risk of developing CRAB infection (*P* < 0.001, Gray test). This factor was also identified as the most influential variable for infection in the Fine–Gray regression model, with an sHR of 1.74 (95% CI: 1.42–2.11; *P* < 0.001). Additional factors identified as associated with CRAB infection were multisite colonization (sHR 1.34; 95% CI: 1.08–1.68; *P* = 0.009), CCI ≥3 (sHR 1.26; 95% CI: 1.01–1.58; *P* = 0.041), male gender (sHR 1.43; 95% CI: 1.12–1.81; *P* = 0.004) and mechanical ventilation (sHR 1.20; 95% CI: 0.98–1.47;, *P* = 0.072), although the latter was not statistically significant (Table 3).

**Table 3.** Multivariable analysis for CRAB infection by Fine–Gray regression

	sHR (95% CI)	*P* value^a^
Multisite colonization^b^	1.34 (1.08–1.68)	**0**.**009**
Charlson comorbidity index ≥3	1.26 (1.01–1.58)	**0**.**041**
Mechanical ventilation^c^	1.20 (0.98–1.47)	0.072
Male gender	1.43 (1.12–1.81)	**0**.**004**
Time to colonization ≤12 d^d^	1.74 (1.42–2.11)	**<0**.**001**

Subdistribution hazard ratios (sHRs) and *P* values from the Fine–Gray regression model for CRAB Infection. Haller C-index: 0.66.

^a^Bold values indicate statistical significance.

^b^Patients with CRAB colonization from more than one specimen from different anatomical sites.

^c^At the time of colonization.

^d^Time from ICU admission to CRAB colonization.

### Model performance: discrimination and calibration

The multivariable logistic regression model for CRAB infection showed moderate discrimination, with an AUROC of 0.70 (95% CI: 0.65–0.75); when the type of critically ill patient was added, the AUROC increased to 0.74 (95% CI: 0.70–0.79). These values were broadly comparable to the discrimination of the Fine–Gray competing risks model (Harrell’s C-index = 0.66). The calibration plot for the primary multivariable logistic model showed good agreement between predicted and observed risk, with only minor deviations from the line of identity and a slight overestimation of risk in the highest-risk decile (Figure S2).

### Impact of different critically ill patients

Table 4 shows the multivariable model adjusted for different types of critically ill patients. In this model, multisite colonization, CCI, mechanical ventilation, male gender and time to colonization were confirmed as independent factors associated with CRAB infection. As for the different critically ill settings, patients following neurosurgery (OR 0.45; 95% CI: 0.23–0.88;, *P* = 0.020) showed a protective effect, whereas those with COVID-19 (OR 2.31; 95% CI: 1.30–4.10; *P* = 0.004) and burn patients (OR 4.84; 95% CI: 1.65–14.17; *P* = 0.004) had a higher risk of CRAB infection. This model had an AUROC of 0.74 (95% CI: 0.70–0.79). Table S4 shows the multivariable model adjusted for a subpopulation without COVID-19 and burns patients.

**Table 4.** Multivariable analysis for CRAB infection adjusted for different types of critically ill patients by logistic regression

	OR (95% CI)	*P* value^a^
Multisite colonization^b^	3.39 (2.25–5.12)	**<0**.**001**
Charlson comorbidity index ≥3	1.70 (1.06–2.73)	**0**.**027**
Mechanical ventilation^c^	1.66 (1.06–2.58)	**0**.**026**
Male gender	1.94 (1.27–2.97)	**0**.**002**
Time to colonization ≤12 d^d^	1.56 (1.04–2.34)	**0**.**031**
Critically ill patients (as categorical)		
General—unspecified^e^	—	**<0.001**
Neurosurgery	0.45 (0.23–0.88)	**0.020**
COVID-19	2.31 (1.30–4.10)	**0.004**
Transplant or CTS	1.28 (0.50–3.24)	0.609
Emergency room patients^f^	1.07 (0.58–1.99)	0.822
Burn patients	4.84 (1.65–14.17)	**0.004**

AUROC 0.74 (95% CI: 0.70–0.79); Hosmer–Lemeshow test: 0.425.

CTS, cardiothoracic surgery unit.

^a^Bold values indicate statistical significance.

^b^Patients with CRAB colonization from more than one specimen from different anatomical sites.

^c^At the time of colonization.

^d^Timing from ICU admission to CRAB colonization.

^e^General—unspecified patients as reference in categorical variable of type of ICU patients.

^f^Unspecified patients admitted in the ER-ICU.

## Discussion

In this study, we found that more than two-thirds of CRAB-colonized patients developed a subsequent CRAB infection in a critical care setting. Multisite colonization, CCI, mechanical ventilation, male gender and early colonization from ICU admission were the key risk factors independently associated with CRAB infection onset. After controlling for competing risks, these predictive factors were confirmed, even if mechanical ventilation was not statistically significant. Additionally, adjusting for different types of critically ill patients, we observed that burn patients or COVID-19 ones had a higher risk of CRAB infection.

To the best of our knowledge, this is the first study to explore predictive factors for CRAB infection in critically ill patients published after the COVID-19 pandemic and the related worldwide nosocomial spread of CRAB.^6^ The infection rate we observed was similar to that reported in 2020 by Qiao and colleagues,^19^ who focused exclusively on critically patients, and higher than that reported in a recent Italian cohort that included general hospitalized patients, not only ICU cases.^20^

Data on prognostic factors in CRAB infection are largely available, and recent studies reported a predictive model for mortality in CRAB BSI or other infections.^21,22^ On the other hand, two interesting studies have explored predictive models for CRAB infection regardless of the previous status of colonization.^9,23^ Of note, the variables included in our analysis reflected some of the most common risk factors for carbapenem-resistant Gram-negative infection, already reported in detailed systemic reviews.^24^

Multisite colonization is one of the most novel findings observed. Although previous colonization is a known risk factor for CRAB infection, the role of multisite colonization was reported recently only in a few single-centre studies focusing on specific types of CRAB infections such as BSI or hospital-acquired pneumonia.^10,11,22,25^ It could be speculated that the amount of bacterial burden may be an important factor in the development of infection. Of note, the role of multisite colonization had been previously validated in a predictive model for Enterobacterales BSI.^26,27^ The confirmation of multisite colonization as a risk factor also in CRAB infection is of great interest in the assessment and management of critically ill patients. Further studies are needed to explore the pathogenetic mechanisms underlying the relation between multisite colonization and CRAB infection onset.

ICU stay has been reported as an infection risk factor in other published studies, but no specific time cut-off has been demonstrated.^24^ Our findings showed a strong association between an early colonization (≤12 days from ICU admission) and CRAB infection onset, as shown in the Kaplan–Meier curve and competing risk analysis. It remains unclear what pathogenic role the time elapsed until colonization plays, whether the risk of CRAB infection is primarily driven by the severity of patients’ condition at ICU admission, or whether additional factors contribute. We could hypothesize that the CRAB isolate may have specific characteristics influencing the time to develop infection, or that in the early phases of colonization the host–microbe equilibrium is unbalanced favouring CRAB replication, leaving doors open for future research in this setting. However, our findings provide a practical cut-off for clinicians, emphasizing the critical role of the time of colonization in assessing and mitigating the risk of CRAB infection.

The role of time to colonization and time from colonization to infection is an open issue. Our finding aligns with observations from a prospective study on KPC-producing *Klebsiella pneumoniae* published in 2022. In that study, Cano *et al.*^28^ demonstrated a temporal association between colonization and infection, suggesting the existence of a ‘high-risk window’ immediately after colonization, during which host susceptibility, pathogen virulence and invasive procedures may converge to increase infection risk. Although the study designs were different, these findings reinforce the concept that not only the presence of colonization, but also its timing, could play a critical role in the pathogenesis of infections caused by MDR organisms and should be considered when designing preventive and therapeutic strategies.

The selected variables in the model include CCI and mechanical ventilation, which are well-known risk factors in CRAB infection.^10,11,24,29^ The CCI cut-off of ≥3 provides a practical cut-off for clinicians, and emphasizes the importance of comorbidity burden in critically ill patients. As for mechanical ventilation, we could not demonstrate an independent association with CRAB infection in the competing risk analysis. Looking at the HR and the lower 95% CI close to 1 (1.20; 95% CI: 0.98–1.47), mechanical ventilation still has a tendency to be a risk factor although not statistically significant, and deserves further investigation.

Finally, in our cohort we found that males had a higher risk of infection. Over the last years, sex differences have become of increasing interest in medicine and critical care research.^30^ Although it is still not clear if sex is or is not a prognostic factor in critically ill patients, it is known that sex differences in physiology, anatomy, immunology and pharmacology could impact management and outcomes in the ICU.^30,31^ Moreover, males are generally more susceptible than females to infections caused by various microorganisms.^32^ Despite our finding, the sex differences in term of risks of infections and outcomes in MDR bacteria should be explored further.

In brief, our findings differ from previous predictive models because they focus on all types of CRAB infection in previously colonized patients in the post-COVID-19 pandemic era.^9–11^ Furthermore, we confirmed the role of well-known risk factors in MDR Gram-negative infection, such as CCI and mechanical ventilation, and highlighted some novel aspects that should be taken into account, such as multisite colonization, time to colonization and sex.^24,29^

Moreover, the combined use of the cumulative incidence function and Fine–Gray regression analysis highlights the importance of accounting for competing risks in critically ill patients.^33^ The model reported here had a reasonable performance and predictive ability, and the predictive factors could be useful to the clinician at the bedside: for instance, a male patient with a CCI score ≥3 (e.g. clinical history of diabetes mellitus with end-organ damage and transient ischaemic attacks), early (fifth day from ICU admission) rectal CRAB colonization, the need for mechanical ventilation, and subsequent respiratory CRAB colonization is at high risk of developing a CRAB infection. In this hypothetical case, if this patient presented signs and symptoms of infection, a CRAB aetiology should be strongly suspected.

Our findings, if validated in other multicentre studies with larger cohorts, could be integrated into a bedside risk score with greater statistical power and superior generalizability than our model. This could be very useful for patients admitted to ICU with CRAB colonization, where the ‘to treat or not to treat’ dilemma is still open, and the balance between antibiotic abuse and prompt and appropriate use remains difficult.^34^

Additionally, we observed how the type of critically ill patient modifies CRAB infection risk. Burn patients (OR 4.84) and COVID-19 ones (OR 2.31) showed markedly higher odds of infection, likely reflecting factors such as extensive skin barrier disruption possibly favouring bacterial translocation, high antibiotic and immunomodulator use, and prolonged mechanical ventilation. Of note, burn patients were found to be at higher risk of CRAB infection in a smaller observational study,^22^ while the COVID-19 result suggests the association between COVID-19 and CRAB infection spread observed in previous studies.^6,7^

Our study has limitations that need to be taken into account when interpreting and applying these results in clinical practice. Firstly, this was an observational study from only two hospitals with several unmeasured variables and residual bias or confounding factors. Notably, infection-control and screening protocols differed between centres and across ICUs, which may have influenced both the likelihood and the timing of detecting colonization and multisite colonization, thereby introducing selection bias. In particular, a dedicated burn unit was present in only one centre and, unlike other ICU patients, burn patients routinely underwent skin swabs, which may have increased the detection of multisite colonization in this subgroup. Secondly, data on the timing of risk factors were not available for all variables, thus we could not analyse all the temporal relationships between colonization, infection and risk factors. We focused our analysis on the risk factors present at the colonization day and the time until the colonization, and handled the competing risks by proper statistical analysis. Thirdly, male gender as a risk factor might reflect unmeasured confounders despite our efforts to avoid it, so this result should be interpreted with caution. Fourthly, time to colonization was derived from surveillance cultures performed at ICU admission and then at regular intervals according to a local screening protocol, rather than continuous sampling, and not all anatomical sites were sampled in all patients. Finally, the inclusion of patients during the COVID-19 pandemic makes it more difficult to generalize our results to settings without SARS-CoV-2 infections. However, avoiding using the COVID-19 variable in the main analysis and performing a sensitivity analysis without COVID-19 and burn patients should have helped us to report findings that could be more generalized in further studies with different epidemiology.

In conclusion, multisite colonization, early colonization, CCI, mechanical ventilation and male gender are independent risk factors for CRAB infection. Burn and COVID-19 patients have a higher risk of infection. Further studies are warranted to validate our findings.

## Supplementary Material

dlaf262_Supplementary_Data

## Data Availability

The data that support the findings of this study are available immediately following publication on request to the corresponding author. The request should be justified with a methodologically sound proposal and approved by the authorship.

## Transparency declarations

M.F. has received unconditional grants from Gilead and speaker’s honoraria from Pfizer, Menarini, Gilead, GSK and ThermoFisher. G.T. has received speaker’s honoraria for educational meetings from Shionogi, honoraria for participation in a scientific board from MSD and speaker’s honoraria from Menarini. A.O. has received speaker’s honoraria for educational meetings from Shionogi, honoraria for participation in a scientific board from Mundipharma and speaker’s honoraria from InfectoPharm. All the reported emoluments are outside this study. All other authors declare no competing interests.

## Supplementary data

Figures S1 and S2 and Tables S1 to S4 are available as Supplementary data at *JAC-AMR* Online.
