# Supplementary material for: Risk factors for carbapenem-resistant Acinetobacter baumannii (CRAB) infections in critically ill patients with previous CRAB colonization: a multicentre cohort study
Source: JAC Antimicrob Resist. 2026 Jan 23;8(1):dlaf262. doi: 10.1093/jacamr/dlaf262 (PMC12828430; doi:10.1093/jacamr/dlaf262)

**Supplementary material**

**Table S1.** STROBE Statement—Checklist of items that should be included in reports of cohort studies

|  | Item No | Recommendation | Page No |
| --- | --- | --- | --- |
| **Title and abstract** | 1 | (*a*) Indicate the study’s design with a commonly used term in the title or the abstract | 1 |
|  |  | (*b*) Provide in the abstract an informative and balanced summary of what was done and what was found | 2 |
| Introduction | | | |
| Background/rationale | 2 | Explain the scientific background and rationale for the investigation being reported | 3 |
| Objectives | 3 | State specific objectives, including any prespecified hypotheses | 4 |
| Methods | | | |
| Study design | 4 | Present key elements of study design early in the paper | 4 |
| Setting | 5 | Describe the setting, locations, and relevant dates, including periods of recruitment, exposure, follow-up, and data collection | 4-5 |
| Participants | 6 | (*a*) Give the eligibility criteria, and the sources and methods of selection of participants. Describe methods of follow-up | 4 |
|  |  | (*b*) For matched studies, give matching criteria and number of exposed and unexposed | NA |
| Variables | 7 | Clearly define all outcomes, exposures, predictors, potential confounders, and effect modifiers. Give diagnostic criteria, if applicable | 5 |
| Data sources/ measurement | 8* | For each variable of interest, give sources of data and details of methods of assessment (measurement). Describe comparability of assessment methods if there is more than one group | 5 |
| Bias | 9 | Describe any efforts to address potential sources of bias | 6-7 |
| Study size | 10 | Explain how the study size was arrived at | 8 |
| Quantitative variables | 11 | Explain how quantitative variables were handled in the analyses. If applicable, describe which groupings were chosen and why | 6 |
| Statistical methods | 12 | (*a*) Describe all statistical methods, including those used to control for confounding | 6-7 |
|  |  | (*b*) Describe any methods used to examine subgroups and interactions | 6-7 |
|  |  | (*c*) Explain how missing data were addressed | 7 |
|  |  | (*d*) If applicable, explain how loss to follow-up was addressed | NA |
|  |  | (*e*) Describe any sensitivity analyses | 7 |
| Results | | |  |
| Participants | 13* | (a) Report numbers of individuals at each stage of study—eg numbers potentially eligible, examined for eligibility, confirmed eligible, included in the study, completing follow-up, and analysed | 8 |
|  |  | (b) Give reasons for non-participation at each stage | 8 |
|  |  | (c) Consider use of a flow diagram | 8 |
| Descriptive data | 14* | (a) Give characteristics of study participants (eg demographic, clinical, social) and information on exposures and potential confounders | 8 |
|  |  | (b) Indicate number of participants with missing data for each variable of interest | 8 |
|  |  | (c) Summarise follow-up time (eg, average and total amount) | 8 |
| Outcome data | 15* | Report numbers of outcome events or summary measures over time | 8 |

**Table S2**. General population and only colonized vs infected patients. Data are number of patients (percentage) except where specified.

| CHARACTERISTICS | general population  n=564 | colonized only patients  N=183 | infected patients  N=381 | OR  (CI 95%) | *p* value |
| --- | --- | --- | --- | --- | --- |
| General, n (%) |  |  |  |  |  |
| Department of hospitalization,  Medical  Surgery  ICU  COVID-19 hospitalization | 128 (22.7)  84 (14.9)  352 (62.4)  192 (34) | 36 (19.7)  32(17.5)  115 (62.8)  44 (24) | 93 (24.1)  52 (13.6)  237 (62.2)  148 (38.8) | 1.3 (0.84-2.00)  0.75 (0.46-1.21)  0.97 (0.68-1.40)  2.01 (1.35-2.98) | 0.235  0.231  0.884  **<0.001** |
| Cause of ICU admission  - septic shock  - respiratory failure  - trauma/politrauma  - cardiogenic shock/cardiac arrest  - neurological disease  - post-surgery  - other | 28 (5)  202 (35.8)  121 (21)  16 (2.8)  84 (14.9)  105 (18.6)  8 (1.4) | 8 (4.4)  51 (27.9)  35 (18.6)  9 (4.9)  37 (20.7)  42 (23)  1 (0.5) | 20 (5.2)  151 (39.6)  86 (22.2)  7 (1.8)  47 (12.3)  63 (16.5)  7 (1.8) | 1.21 (0.56-2.81)  1.7 (1.16-2.49)  1.25 (0.81-1.94)  0.36 (0.13-0.99)  0.56 (0.35-0.90)  0.66 (0.42-1.03)  3.44 (0.42-28.2) | 0.004^°^ |
| In previous 90 days from admission, n (%) |  |  |  |  |  |
| Hospitalization | 128 (22.7) | 39 (21.3) | 89 (23.4) | 1.14 (0.75-1.73) | 0.548 |
| ICU admission* | 45 (8) | 15 (8.2) | 30 (7.9) | 0.94 (0.50-1.77) | 0.908 |
| Antibiotic therapy | 181 (32.1) | 55 (30.1) | 126 (33.1) | 1.15 (0.8-1.71) | 0.473 |
| MDRO infection* | 56 (10) | 17 (9.3) | 39 (10.3) | 1.12 (0.31-2.04) | 0.711 |
| Gram-negative MDR infection* | 23 (4.1) | 10 (5.5) | 13 (3.5) | 0.622 (0.27-1.45) | 0.266 |
| Immunosuppression¨ | 128 (22.7) | 39 (21.3) | 89 (23.4) | 1.12 (0.72-1.72) | 0.587 |
| Previous surgery  of which abdominal  neurosurgery  cardiac surgery  orthopaedic  others (75% burn-related) | 255 (45.2)  59 (23.2)  83 (32.7)  16 (6.3)  43 (16.9)  53 (20.6) | 94 (51.4)  21 (21.5)  44 (47.3)  6 (6.5)  11 (11.8)  12 (12.9) | 161 (42.3)  39 (24.2)  39 (24.2)  10 (6.2)  32 (19.9)  41 (25.5) | 0.69 (0.48-0.99)  1.12 (0.62-2.09)  0.36 (0.21-0.61)  0.96 (0.31-2.29)  1.74 (0.85-3.56)  2.31 (1.14-4.65) | **0.042**  0.621  **<0.001**  0.736  0.099  **0.018** |
| Comorbidities, n (%) |  |  |  |  |  |
| Hypertension | 246 (43.7) | 73 (39) | 175 (43.7) | 1.33 (0.93-1.9) | 0.122 |
| Obesity (BMI ≥ 30)* | 72 (13.4) | 22 (12.6) | 50 (13.7) | 1.2 (0.62-1.79) | 0.736 |
| Peripheral vascular disease | 48 (8.5) | 14 (7.7) | 34 (8.4) | 1.10 (0.59-2.03) | 0.625 |
| Cerebrovascular disease | 56 (9.9) | 21 (11.5) | 35 (9.2) | 0.79 (0.44-1.39) | 0.408 |
| Dementia | 17 (3) | 5 (2.7) | 12 (3.1) | 1.17 (0.4-3.36) | 0.774 |
| Chronic obstructive pulmonary disease | 49 (8.7) | 12 (6.6) | 37 (9.7) | 1.59 (0.81-3.0) | 0.220 |
| Connective tissues diseases | 12 (2.1) | 5 (2.7) | 7 (1.8) | 0.66 (0.21-2.12) | 0.779 |
| Peptic ulcer disease | 8 (1.4) | 1 (0.5) | 7 (1.8) | 3.43 (0.42-28.13) | 0.227^ |
| Liver disease ^1^ | 19 (3.4) | 6 (3.2) | 13 (3.4) | 1.05 (0.39-2.8) | 0.925 |
| Hemiplegia | 23 (4) | 10 (5.5) | 13 (3.4) | 0.61 (0.26-1.4) | 0.255 |
| HIV infection | 7 (1.2) | 2 (1.1) | 5 (1.3) | 1.22 (0.23-6.33) | 0.815 |
| Autoimmune disease | 23 (4.1) | 10 (5.5) | 13 (3.4) | 0.64 (0.29-1.43) | 0.279 |
|  |  |  |  |  |  |

*Legend*: ICU: Intensive Care Unit; MDR: Multi-Drug Resistance; MDRO: Multi-Drug Resistance Organism; BMI: Body Mass Index; {1} from chronic hepatitis to cirrhosis

*Missing data. Total patient all items of Obesity: n 539; Previous 90d data: n 562

° Global p-value for Type of ICU patients (χ² test).

^fisher test

¨immunosuppression: at least one between immunotherapy (TNF-α inhibitors, cyclophosphamide, azathioprine, methotrexate or mycophenolate mofetil), steroids (prednisone (or its equivalent) at a dose of >0.5 mg/kg/day for at least 1 month), chemotherapy

**Table S3.** Multivariable analysis for CRAB infection by logistic regression to explore the impact of centre-specific screening strategy.

|  | **OR (95% CI)** | **P value** |
| --- | --- | --- |
| **Multisite colonization^1^** | 2.93 (1.99 – 4.32) | **<0.001** |
| **Charlson Comorbidity Index ≥ 3** | 1.58 (1.00 – 2.49) | **0.052** |
| **Mechanical ventilation^2^** | 1.35 (0.89 – 2.06) | 0.156 |
| **Male gender** | 2.08 (1.39 – 3.13) | **<0.001** |
| **Timing to colonization ≤ 12 d^3^** | 1.98 (1.34 – 2.93) | **<0.001** |
| **IPC protocol^4^** | 0.78 (0.51-1.20) | 0.242 |

AUROC 95% CI: 0.70 (0.66-0.75), Hosmer-Lemeshow test: 0.019

*Legend*: 1: patients with CRAB colonization from more than one specimen from different anatomical sites; 2: at the time of colonization; 3: timing from ICU admission to CRAB colonization; 4: infection prevention and control protocol that differs between the two centers.

**Table S4.** Multivariable analysis for CRAB infection by logistic regression in a subpopulation without COVID-19 and burn patients (total num. 347, 210 infected vs 136 only colonized).

|  | **OR (95% CI)** | **P value** |
| --- | --- | --- |
| **Multisite colonization^1^** | 3.53 (2.18 – 5.70) | **<0.001** |
| **Charlson Comorbidity Index ≥ 3** | 2.24 (1.26 – 3.97) | **0.006** |
| **Mechanical ventilation^2^** | 1.25 (0.78 – 2.00) | 0.354 |
| **Male gender** | 2.03 (1.23 – 3.36) | **0.006** |
| **Timing to colonization ≤ 12 d^3^** | 1.92 (1.20 – 3.01) | **0.007** |

AUROC 95% CI: 0.71 (0.66-0.77), Hosmer-Lemeshow test: 0.595

*Legend*: 1: patients with CRAB colonization from more than one specimen from different anatomical sites; 2: at the time of colonization; 3: timing from ICU admission to CRAB colonization.

**Figure S1.** Classification and Regression Trees (CART) analysis according to their association with CRAB infection onset focusing on time to colonization.

**Figure S2.** Calibration plot of the multivariable logistic regression model for CRAB infection in colonised ICU patients


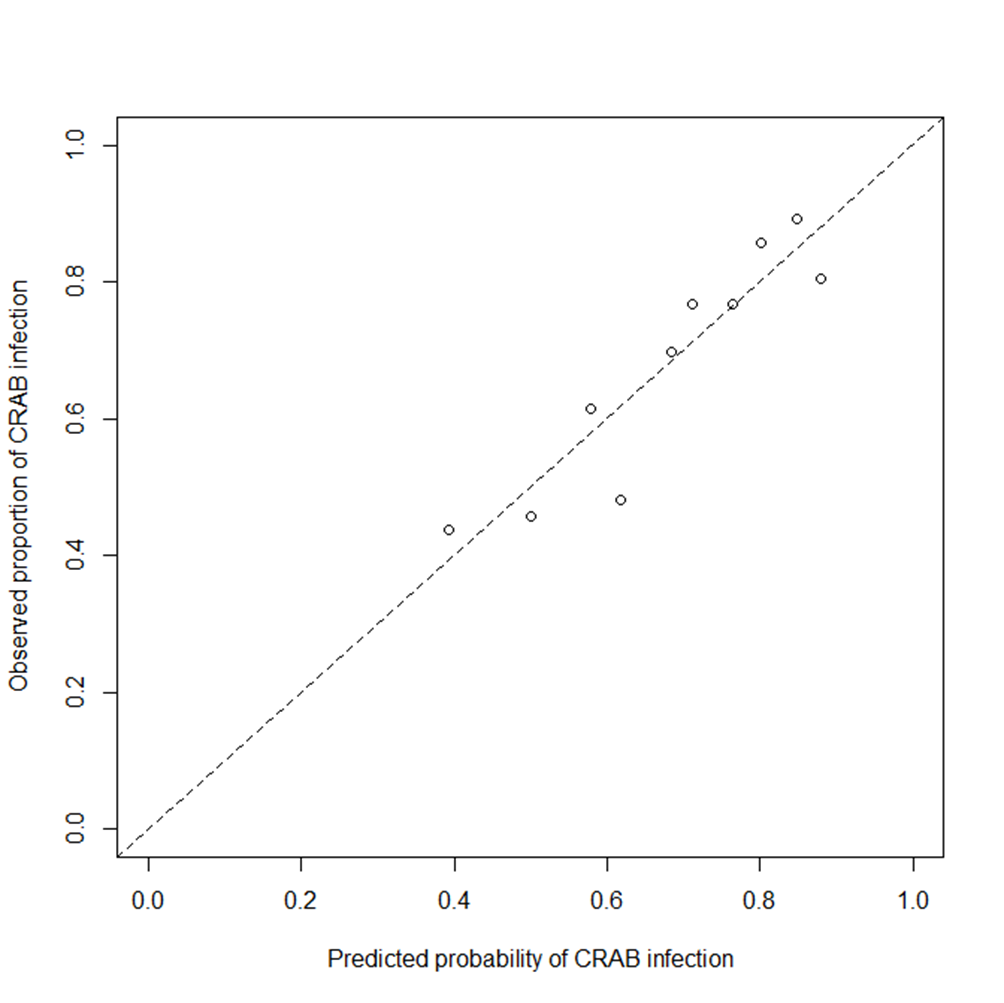

Supplement: dlaf262_Supplementary_Data [file dlaf262_supplementary_data.docx]
